# Investigation of image-based lesion and kidney dosimetry protocols for ^177^Lu-PSMA-I&T therapy with and without a late SPECT/CT acquisition

**DOI:** 10.1186/s40658-023-00529-8

**Published:** 2023-02-09

**Authors:** Sandra Resch, Sarah Takayama Fouladgar, Mathias Zacherl, Gabriel T. Sheikh, Grigory Liubchenko, Mikhail Rumiantcev, Lena M. Unterrainer, Vera Wenter, Peter Bartenstein, Sibylle I. Ziegler, Harun Ilhan, Leonie Beyer, Guido Böning, Astrid Delker

**Affiliations:** Department of Nuclear Medicine, University Hospital, LMU Munich, Munich, Germany

**Keywords:** SPECT, PSMA, ^177^Lu, Prostate cancer, Dosimetry

## Abstract

**Background:**

^177^Lu-PSMA therapy has been successfully used to prolong the survival of patients with metastatic castration-resistant prostate cancer. Patient-specific dosimetry based on serial quantitative SPECT/CT imaging can support the understanding of dose–effect relationships. However, multiple SPECT/CT measurements can be challenging for patients, which motivates the investigation of efficient sampling schedules and their impact on dosimetry. In this study, different time samplings with respect to the number and timing of SPECT/CT acquisitions with and without a late measurement were investigated.

**Materials and methods:**

In total, 43 lesions and 10 kidneys of 5 patients receiving ^177^Lu-PSMA-I&T therapy were investigated. Whole-body SPECT/CT measurements were performed at 1, 2, 3 and 7 days post-injection. For both lesions (isocontour-based segmentation) and kidneys (CT-based segmentation), a reference model was employed including all four time points. To identify the best-matching fit function out of a pre-defined set of models, visual inspection, coefficients of variation and sum of squared errors were considered as goodness-of-fit criteria. Biologically effective doses (BEDs) calculated with different time samplings (days 1, 2, 3/1, 2, 7/1, 3, 7/2, 3, 7 and 1, 2/1, 3/1, 7) were compared to the reference.

**Results:**

The best-fit function was found to be a mono-exponential model for lesions and a bi-exponential model with a population-based parameter and two free parameters for kidneys. The BEDs calculated with the time sampling 1, 3, 7 days showed the lowest deviations from the reference for lesions with 4 ± 5%. Without day 7, still 86% of all lesions showed deviations from the reference < 10%. The outlier deviations showed a positive correlation with the effective half-life of the respective lesions. For kidneys, including days 1, 2, 3 achieved the best results with 0 ± 1%. Generally, deviations for kidneys were found to be small for all time samplings (max. 13%).

**Conclusions:**

For combined optimization of the SPECT/CT time sampling for kidney and lesion dosimetry during ^177^Lu-PSMA-I&T therapy, the sampling with days 1, 3, 7 showed the smallest deviation from the reference. Without a late acquisition, using the schedule with days 1, 2, 3 is likewise feasible.

**Supplementary Information:**

The online version contains supplementary material available at 10.1186/s40658-023-00529-8.

## Introduction

Prostate cancer still is the most common type of cancer in men worldwide [1]. Once metastases have developed, the 5-year relative survival decreases from 100 to 31% [2]. However, for late-stage metastatic castration-resistant prostate cancer (mCRPC), radioligand therapy targeting the prostate-specific membrane antigen (PSMA) has proven to decrease prostate-specific antigen levels in the body. This can be associated with an increase in the overall survival and an improvement in the quality of life [3, 4]. Therefore, in March 2022, the FDA approved the first radioactive compound targeting mCRPC, namely Pluvicto™ (Novartis AG), formerly known as ^177^Lu-PSMA-617 [5]. Furthermore, a clinical study has been started in February 2022 investigating the compound ^177^Lu-PSMA-I&T for mCRPC [6].

Recently, the European Association of Nuclear Medicine has published guidelines covering internal dosimetry of ^177^Lu-labeled PSMA ligands with regard to skin, bone marrow and blood, glands, kidneys and tumors [7]. Sequential hybrid planar–SPECT/CT or SPECT/CT acquisitions are recommended with one to three measurements ranging from day 1 up to day 7 post-injection (p.i.) for kidneys and three to five time points (TPs) for tumors. For the latter, the last acquisition should lie between 3 and 7 days p.i. with a tendency toward later TPs [8]. These guidelines also indicate that it is not only important to monitor the absorbed dose to organs-at-risk over the course of the treatment, but also to investigate treatment response in the form of tumor-absorbed doses. Specifically, the biologically effective dose (BED) can be correlated with treatment outcomes such as tumor volume reduction or changes in the PSA levels, offering the potential for an improved therapy [9, 10]. It has been shown in ^177^Lu-DOTATATE therapy that there is a long-term tumor retention which leads to measurable activity signal even 5 to 7 weeks p.i., suggesting the importance of measurements later than 7 days after therapy [11]. However, for ^177^Lu-PSMA, the impact of late imaging TPs on tumor dosimetry for patients has not been investigated explicitly [8].

Looking at current clinical imaging protocols after ^177^Lu-PSMA therapy, mainly whole-body planar scintigraphies or combinations of planar acquisitions and SPECT/CT are acquired and the most commonly used TPs are 4 h, 1, 3, 4 and 7 days p.i. with mostly three or four measurements in total [12–17]. Rinscheid et al. investigated the optimization of sampling schedules comprising one to four measurements out of 24 possible TPs ranging from 1 to 192 h using a hybrid planar/SPECT approach. The study is based on 13 patients whose biokinetic data were used to create virtual TACs of two kidneys and two lesions using physiologically based pharmacokinetic modeling and artificial noise. The noiseless TACs served as ground truth. They found out that the smallest root-mean-squared error (RMSE) of the deviation of the tumor- and kidney-TACs from the ground truth, i.e., the best sampling, was achieved by including three TPs at 3–4, 96–100 and 192 h p.i. with similar results when four TPs in that range were included. If the last imaging TP was set to 120 h p.i., the RMSE of the tumors was still less than 10% [18]. Regarding the choice of the best-fit function, Hardiansyah et al. investigated a total amount of 20 fit functions for the renal biokinetics of 13 patients for ^177^Lu-PSMA-I&T therapy [19]. They found a bi-exponential function with a population-based, shared parameter to be the best-fit function according to the model selection method developed by Kletting et al. [20]. More research is needed to establish a standardization of SPECT/CT TPs and warrant recommendations on which TAC fit model is appropriate to use particularly for tumor dosimetry after ^177^Lu-PSMA radioligand therapy. In Germany, patients who received ^177^Lu-PSMA therapy can be released from the hospital 2–3 days p.i. and examinations later than 3 days must be performed on an outpatient basis. In other countries, all scans are performed on an outpatient basis and patients have to travel back and forth from the hospital. Every scan that could be avoided would reduce logistic effort and improve patient compliance. This motivates a deeper analysis of this topic.

Thus, the aim of this study was to evaluate the impact of time sampling and TAC fit model on the lesion and kidney BED for ^177^Lu-PSMA-I&T toward clinically feasible dosimetry protocols.

## Materials and methods

### Phantom measurement

Phantom measurements, using the NEMA IEC body phantom, were conducted to assess the general feasibility of fast SPECT imaging over an extended field-of-view, using a projection time of 5 s in combination with the clinically used imaging and reconstruction parameters. Additionally, the threshold for a reasonable lesion volume should be determined for this study. All six spheres (26.52, 11.49, 5.58, 2.57, 1.15 and 0.52 ml) were filled with an activity concentration of 1046 kBq/ml and the background was filled with 65 kBq/ml, resulting in a sphere-to-background ratio of 16:1. This high ratio was chosen because mainly bone lesions were evaluated, which are typically located in a low background activity. The sphere recovery coefficients were determined by dividing the respective average activity concentration as measured in the SPECT by the known activity concentration. The background activity was not considered in these calculations. To measure the activity concentration in the spheres, two methods were used, a CT-based and a threshold-based method. In the CT-based method, spherical volumes-of-interest (VOIs) with the known sphere diameters were placed on the high-resolution CT images (voxel size 1.2 × 1.2 × 1.2 mm^3^) and thereafter transferred to the SPECT images to collect the mean activity concentration in the VOIs. However, defining bone lesions in clinical low-dose CT images may be challenging. Thus, in the second method, the spheres were delineated directly on the SPECT images using a threshold-based method originally evaluated on PET images [21]. In this method, a spherical VOI with a 12 mm diameter was employed with the sphere center being placed at the maximum activity concentration of each lesion. A 30%-isocontour of the average within this VOI was then used to obtain the respective lesion contours.

### Patients

This study is based on five patients with mCRPC, who underwent their first cycle of ^177^Lu-PSMA-I&T therapy. PSMA expression was verified prior to therapy via ^18^F-PSMA-1007 PET/CT imaging. All patients gave written consent to undergo radioligand therapy. All data were irreversibly anonymized before evaluation. The study was performed in a retrospective manner with approval from the local ethics committee (project number: 22-0552). Four patients were administered a therapeutic activity of 7417 ± 22 MBq, while one patient received 8925 MBq ^177^Lu-PSMA-I&T due to diffuse bone metastases. For kidney protection, the patients received an infusion of 1000 ml of NaCl shortly after therapy and were instructed to ensure adequate hydration. SPECT/CT measurements were acquired at 19 ± 1 h, 42 ± 1 h, 66 ± 1 h and 170 ± 2 h p.i (days 1, 2, 3 and 7 in the following) over three bed positions beginning from the eyes downward. Within the clinical protocol at our department, no measurements before 1 day p.i. are acquired for logistic reasons and patient comfort on the day of therapy [17]. The acquisition time for the complete SPECT field-of-view of about 1.2 m was approximately 15 min. In four patients, metastases were mainly localized in the skeleton, whereas one patient predominantly had lymph node metastases.

### Image acquisition and reconstruction

Quantitative SPECT images were acquired for both, the patients and the phantom, on a dual-headed Siemens Symbia Intevo T16 SPECT/CT (Siemens Healthineers, Erlangen, Germany) using the upper photopeak of ^177^Lu (208 keV, width: 15%) with scatter windows (170 keV, width 15% and 240 keV, width 10%) and the medium-energy low-penetration collimator. According to the standard clinical protocol, the matrix size was set to 128 × 128 pixels. 64 projections per detector head were acquired in auto-contour mode, each with an acquisition time of 5 s. Quantitative SPECT images were reconstructed with Hermes Hybrid Recon-Oncology 4.0 (Hermes Medical Solutions, Sweden), using a calibration factor of 7.9 cps MBq^−1^, a maximum-a-posteriori ordered-subset expectation–maximization (MAP-OSEM) algorithm with 16 iterations, 8 subsets, and a quadratic penalty with a factor of *β* = 0.001. Quantitative reconstruction included CT-based attenuation correction, a Monte Carlo-based scatter correction, and resolution modeling [22, 23]. The CT measurement was conducted with 110 keV tube voltage and with CareDose enabled using a reference current of 15 mAs and a slice thickness of 3 mm.

### Image processing

Further processing of the reconstructed clinical SPECT images was continued in PMOD (Version 3.609, PMOD TECHNOLOGIES LLC, Switzerland). The lesions were segmented day-wise according to the iso-contour method described in the phantom section. Kidney VOIs were drawn manually slice by slice on the CT images. The mean activity concentration and the VOI volume were extracted from PMOD for each lesion and kidney. Further, to correct absorbed doses for the individual VOI mass, for each VOI the corresponding Hounsfield Unit (HU) was extracted from the CT data and converted into an average VOI density. In order to convert the mean HU of each VOI into density values, a calibration curve was employed, which was previously established via a Gammex Tissue Phantom [24]. More Precisely, the known densities of 11 tissue-mimicking materials were plotted against the HU as measured for the employed CT system and fitted by a linear model, resulting in *ρ* = 7.94E−5 ± 0.34E−5*HU + 9.64E−1 ± 0.18E−1. This provides a formula to convert the mean HU of each lesion or kidney VOI into a mean density. The respective plot with linear fitting is shown in Additional file 1. In total, 43 lesions, five of which were soft tissue and the rest bone lesions, and 10 kidneys were segmented.

### Fit models and model selection

In MATLAB (v2011-R2016), the absorbed dose of each VOI was calculated. In order to obtain the time-integrated activity (TIA), the TAC model was fitted to the VOI data using a nonlinear-least-squares algorithm and integrated from zero to infinity. For the determination of the reference model for both, kidneys and lesions, all four available TPs (1, 2, 3 and 7 days p.i.) were included and the ‘best’ fit function was evaluated. All models proposed by Hardiansyah et al. were considered, which were originally evaluated for kidney biokinetics [19]. The goodness-of-fit was checked by visual inspection, the coefficient of variation (CV) in percent and the sum of squared errors (SSE) averaged over all lesions [19, 20, 25]. The CV includes the standard error of the fit parameters which should optimally be small. CVs less than 25% usually indicate that the parameter estimates are precise. Since all tested fit functions have the same degrees of freedom, the SSE provides information about the quality of the model: the smaller the SSE, the smaller the difference between the data point and model estimate.

After a first selection process, which removed all functions for which the fitting either failed or had coefficients of variation (CV) larger than 100%, the following functions were further considered for model selection:

#### For lesions:

$$f_{{{\text{mono}}}} \left( t \right) = A_{0} e^{{ - \left( {\lambda_{{{\text{bio}}}} + \lambda_{{{\text{phys}}}} } \right)t}} ,$$

$$f_{{{\text{bi}}1,{\text{s}}\upgamma }} \left( t \right) = A_{0} {\varvec{\gamma}}e^{{ - \left( {\lambda_{{{\text{bio}}}} + \lambda_{{{\text{phys}}}} } \right)t}} + A_{0} \left( {1 - {\varvec{\gamma}}} \right)e^{{ - \left( {\lambda_{{{\text{phys}}}} } \right)t}} \;{\text{with}}\;{\text{shared}}\;\gamma ,$$

$$f_{{{\text{bi}}1,{\text{s}}{\varvec{\lambda}}}} \left( t \right) = A_{0} \gamma e^{{ - \left( {{\varvec{\lambda}}_{{{\mathbf{bio}}}} + \lambda_{{{\text{phys}}}} } \right)t}} + A_{0} \left( {1 - \gamma } \right)e^{{ - \left( {\lambda_{{{\text{phys}}}} } \right)t}} \;{\text{with}}\;{\text{shared}}\;\lambda_{{{\text{bio}}}} .$$

#### For Kidneys:

$$f_{{{\text{mono}}}} \left( t \right) = A_{0} e^{{ - \left( {\lambda_{{{\text{bio}}}} + \lambda_{{{\text{phys}}}} } \right)t}} ,$$

$$f_{{{\text{bi}}1,{\text{s}}\upgamma }} \left( t \right) = A_{0} {\varvec{\gamma}}e^{{ - \left( {\lambda_{{{\text{bio}}}} + \lambda_{{{\text{phys}}}} } \right)t}} + A_{0} \left( {1 - {\varvec{\gamma}}} \right)e^{{ - \left( {\lambda_{{{\text{phys}}}} } \right)t}} \;{\text{with}}\;{\text{shared}}\;\gamma ,$$

$$f_{{{\text{bi}}1,{\text{s}}{\varvec{\lambda}}}} \left( t \right) = A_{0} \gamma e^{{ - \left( {{\varvec{\lambda}}_{{{\mathbf{bio}}}} + \lambda_{{{\text{phys}}}} } \right)t}} + A_{0} \left( {1 - \gamma } \right)e^{{ - \left( {\lambda_{{{\text{phys}}}} } \right)t}} \;{\text{with}}\;{\text{shared}}\;\lambda_{{{\text{bio}}}} ,$$

and

$$f_{{{\text{bi}}2,{\text{sA}}1}} \left( t \right) = A_{0} e^{{ - \left( {\lambda_{{{\text{bio}}}} + \lambda_{{{\text{phys}}}} } \right)t}} + {\varvec{A}}_{1} e^{{ - \left( {\lambda_{{{\text{phys}}}} } \right)t}} \;{\text{with}}\;{\text{shared}}\;A_{1} ,$$

with the activities *A*_0_ (*A* (*t* = 0)) and *A*_1_, the factor *γ* (0 < *γ* < 1), the physical decay constant *λ*_phys_ = 4.34E−3 h^−1^ of ^177^Lu and the biological clearance rate *λ*_bio_. The mono-exponential function is characterized by two free parameters, *A*_0_ and *λ*_bio_, whereas the bi-exponential functions use additional parameters *γ* and *A*_1_. For all bi-exponential functions, one parameter each is considered as a population-based, shared parameter. They are estimated using the Jackknife method [26, 27]. Within this approach, the shared parameter is determined for each patient by fitting the respective bi-exponential function to all lesions from the patient cohort, with all lesions from the patient under consideration being excluded. An exception was made for the kidney *γ*-factor, which has already been reported to be 0.963 ± 0.004 for a large population of 13 ^177^Lu-PSMA-I&T patients [19]. This procedure reduces the number of free parameters for the bi-exponential functions to two. Out of the functions presented above, the best-fit function was chosen to be the one with the smallest CV and SSE separately for lesions and kidneys.

### Biologically effective dose calculation

The absorbed dose was estimated following the MIRD scheme with the assumption that the source region is equal to the target region due to the short range of beta radiation from the ^177^Lu decay [28]. Using the time-integrated activity (TIA), the absorbed dose D can be determined by

$$D = {\text{TIA}} \cdot S$$

with the *S*-factor being the mean absorbed dose to the target region per radioactive decay in the source region. The used lesion *S*-value of 2.33E−5 Gy MBq^−1^ s^−1^ (for 1 g tumor) was derived from OLINDA/EXM with the approximation of unit density spheres corrected for the lesion-specific average density [29]. The kidney *S*-value of 7.38E−8 Gy MBq^−1^ s^−1^ (for 310 g kidneys) was likewise corrected for the patient-specific kidney mass [30].

In the next step, the BED was derived using the equation

$${\text{BED}} = D \cdot {\text{RE}}$$

with the relative effectiveness RE. For radioligand therapy, the RE can be written as

$${\text{RE}} = 1 + \frac{D}{\alpha /\beta } \cdot G,$$

where *α* and *β* are radiosensitivity constants defined by the tissue and G is called the Lea-Catcheside factor [31]. Assuming a mono-exponentially decreasing activity in the source organ, *G* is expressed as

$$G = \frac{{\lambda_{{{\text{eff}}}} }}{{\mu + \lambda_{{{\text{eff}}}} }} ,$$

where *λ*_eff_ = *λ*_phys_ + *λ*_bio_ are the effective clearance rate of the source region and *µ* is the repair rate of the tissue. In case of a bi-exponential clearance, the Lea-Catcheside-factor is given by

$$G = \frac{{\frac{{a_{1}^{2} }}{{\lambda_{1} \left( {\mu + \lambda_{1} } \right)}} + \frac{{2a_{1} a_{2} }}{{\left( {\lambda_{1} + \lambda_{2} } \right)\left( {\mu + \lambda_{1} } \right)}} + \frac{{2a_{2} a_{1} }}{{\left( {\lambda_{2} + \lambda_{1} } \right)\left( {\mu + \lambda_{2} } \right)}} + \frac{{a_{2}^{2} }}{{\lambda_{2} \left( {\mu + \lambda_{2} } \right)}}}}{{\left( {\frac{{a_{1} }}{{\lambda_{1} }} + \frac{{a_{2} }}{{\lambda_{2} }}} \right)^{2} }}$$

with λ_1_ = λ_eff_ and λ_2_ = λ_phys_ [32]. The so-called dose rate fraction coefficients a_1_ and a_2_ are hereby determined as follows:

$$a_{1} = \frac{{A_{0} \cdot \gamma }}{{f_{{{\text{bi}}}} \left( {t = 0} \right)}}\;{\text{and}}\;a_{2} = \frac{{A_{0} \left( {1 - \gamma } \right)}}{{f_{{{\text{bi}}}} \left( {t = 0} \right)}} .$$

The *α*/*β*-ratio of tumors was set to *α*/*β* = 3.1 Gy as reported by Wang et al. for mCRPC [33]. For the repair rate, a repair half-life *T*_*µ*_ of 1.9 h was used [33]. The respective kidney parameters were *α*/*β* = 2.6 Gy and *T*_*µ*_ = 2.8 h [34].

In addition to lesion-wise calculation, total tumor dose and respective BED were determined by averaging the activity concentrations and summing the tumor masses of all lesions of one patient.

### Data evaluation

In order to evaluate the impact of different time samplings, the BED was calculated for all lesions and kidneys with different sampling schedules and compared to the reference model. The fit model determined for the reference was also used for all other time samplings. Since in many countries every SPECT/CT measurement has to be performed on an outpatient basis and every way back and forth from the hospital may be challenging for the patients, the following sets of data points were investigated: days 1, 2, 3 versus 2, 3, 7 versus 1, 2, 7 versus 1, 3, 7 with all including three TPs and days 1, 2 versus 1, 3 versus 1, 7 comprising two TPs. All possible variations of three TPs were used while for two TPs, it is assumed that patients stay at the hospital until 1 day p.i. and just have to come in again once for the second measurement.

The performance of these time samplings regarding dosimetry was evaluated by Bland–Altman plots and relative deviations (RD) from the respective reference model:

$${\text{RD}} = \frac{{{\text{BED}} - {\text{BED}}_{{{\text{reference}}}} }}{{{\text{BED}}_{{{\text{reference}}}} }} \cdot 100\% .$$

Thus, positive RD indicates an overestimation of the BED compared to the reference, and negative RD an underestimation. In order to assess the total deviation of all BEDs from the reference model within one sampling schedule, the absolute deviation (MD) was calculated and averaged over all lesions or kidneys.

## Results

### Phantom measurement

**Fig. 1:**
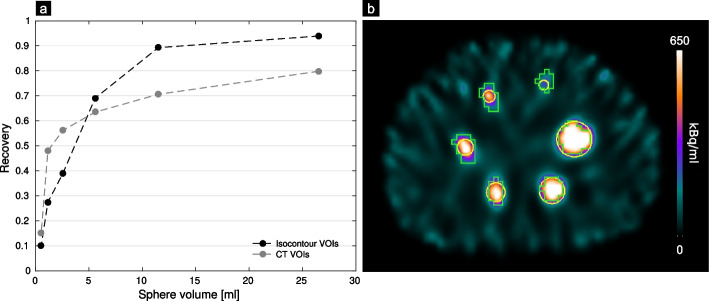
In **a**, the recovery coefficients are plotted against the volumes of all spheres (0.52, 1.15, 2.57, 5.58, 11.49 and 26.52 ml) of the NEMA IEC Body Phantom filled with a sphere-to-background ratio of 16:1 (total activity of 680 MBq), which was imaged with 5 s per projection. Recovery coefficients are provided for CT-based (yellow) and isocontour-based sphere segmentation (green) shown in **b**

The recovery curves and corresponding phantom VOIs for the six spheres of the NEMA IEC Body Phantom for CT-based and isocontour-based segmentation are demonstrated in Fig. 1a, b. The mean recovery coefficients of the largest three spheres were higher for isocontour VOIs than for CT VOIs with maximum values of 0.94 and 0.80, respectively. The behavior was different for the three smallest spheres, where the recovery of the CT VOIs was higher. For the next steps, i.e., the patient measurements and corresponding lesion evaluations, the phantom analysis indicated high recoveries for 5 s exposure time per projection. However, the segmented spheres with a volume below 5 ml showed a high deviation from the true volume. The isocontour-based method overestimates the volumes of the three smallest spheres by factors of 2.7, 4.9 and 5.9 for decreasing sphere volume. This could lead to errors in the BED calculation. Therefore, further analysis of patient data was performed with a volume limit of 5 ml for isocontour-based lesion segmentation.

### Patients

**Fig. 2:**
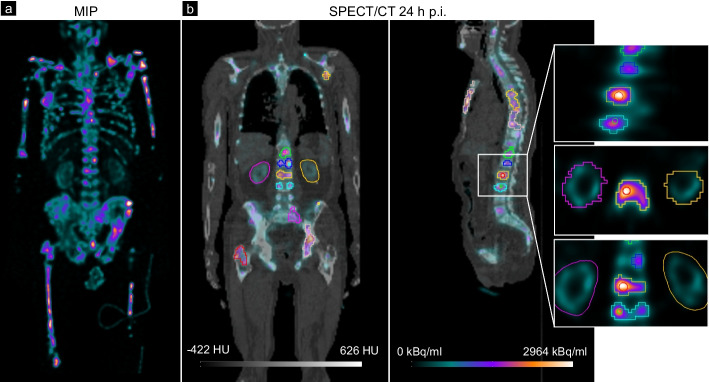
In **a**, the maximum intensity map (MIP) of the SPECT/CT of patient 1 at 1 day p.i. is visualized, illustrating the high tumor load of this patient. In the fused SPECT and CT images in **b**, the segmentations of exemplarily selected lesions (30% threshold of average in 12-mm sphere placed at the lesion maximum) and both kidneys (manual segmentation in the coronal plane) are highlighted

Table 1 summarizes the main data of the five patients included in this study. As expected, the total tumor BED of 13.7 Gy is the largest for patient 1, since this patient was treated with the highest activity of approximately 9 GBq. Further, patient 1 shows the largest segmented total tumor volume of 569.9 ml and the highest segmented single tumor volume of 121.0 ml. For patient 4, only four soft tissue lesions could be segmented which still sum up to a total tumor volume of 119.0 ml. The SPECT/CT data acquired at 1 day p.i. for patient 1 are illustrated exemplarily in Fig. 2.

**Table 1:** Summary of the evaluated patient data. The total tumor BED was calculated for all segmented lesions per patient using the mono-exponential fit model and all available data points (days 1, 2, 3 and 7)

Patient	Injected activity [GBq]	Number of seg. lesions	Total tumor BED_3.1 Gy_ [Gy]	Max. tumor BED_3.1 Gy_ [Gy]	Total tumor volume [ml]	Mean tumor volume [ml] (min., max.)
1	8.9	15	12.8	21.7	569.9	33.9 (10.1, 121.0)
2	7.4	7	5.6	15.1	73.7	10.5 (5.0, 29.2)
3	7.4	8	5.1	11.5	340.2	42.5 (17.2, 96.7)
4	7.4	4	7.2	10.8	119.0	29.7 (9.3, 78.0)
5	7.4	9	4.0	5.4	96.9	10.7 (5.6, 20.3)

### Evaluation of the reference model

After the exclusion of all fit functions for which the fit failed or the CV was larger than 100%, the remaining functions and their goodness-of-fit parameters are summarized in Table 2. All fitted lesion TACs are provided in Additional file 1. For the lesions, the mono-exponential model *f*_mono_ performed best. However, the bi-exponential model with a shared *γ* parameter visually performed equally well. The MD of the BEDs calculated with this function (*f*_bi1,sγ_) from the reference model *f*_mono_ is 23 ± 16%.

**Table 2:** Summary of the goodness-of-fit parameters and the shared population-based parameters for the evaluated lesion and kidney fit functions

Fit function	Mean CV [%] (max)		Mean SSE ± SD	Mean population parameter ± SD	MD of BED [%] ± SD (max)
	*P*_1_	*P*_2_			
*Lesions*					
***f***_**mono**_	7.5 (15.4)	14.3 (26.5)	9.8 ± 18.3	–	–
*f*_bi1,sγ_	9.7 (30.0)	17.4 (45.7)	12.2 ± 30.7	0.799 ± 0.023 (*γ*)	23 ± 16 (69)
*f*_bi1,sλ_	9.4 (24.2)	20.5 (55.6)	34.3 ± 102.6	0.013 ± 0.006 h^−1^ (*λ*_bio_)	21 ± 11 (40)
*Kidneys*					
*f*_mono_	3.6 (10.5)	5.3 (17.4)	6.1 ± 4.3	–	9 ± 3 (15)
***f***_**bi1,sγ**_	3.2 (10.3)	3.9 (14.0)	3.7 ± 3.5	0.963 ± 0.004 (*γ*)*	–
*f*_bi1,sλ_	4.2 (9.1)	3.9 (8.0)	20.1 ± 19.3	0.021 ± 0.001 h^−1^ (*λ*_bio_)	12 ± 12 (26)
*f*_bi2,sA1_	4.2 (10.8)	4.8 (13.2)	6.3 ± 7.9	6.220 ± 1.019 MBq (*A*_2_)	4 ± 4 (10)

*P*_1_ and *P*_2_ refer to the respective two free parameters of each function. The functions for which the fit failed or the maximum CV was above 100% were neglected for model selection. The best-fit functions are highlighted by bold font. (*CV* coefficient of variation, *SSE* sum of squared errors, *SD* standard deviation, *MD* mean absolute deviation)

*Parameter value according to Hardiansyah et al. [19]

All TAC fits of the kidney data resulted in very similar goodness-of-fit parameters except the high SSE for the *f*_bi1,sλ_ model. The function *f*_bi1,sγ_ was chosen as the reference function, since it has low CVs for both parameters and the smallest SSE. Using the kidney data available in this study resulted in a population-based value of 0.952 ± 0.009 for the shared parameter *γ*.

### Influence of the time sampling on the BED

#### Lesions

**Fig. 3:**
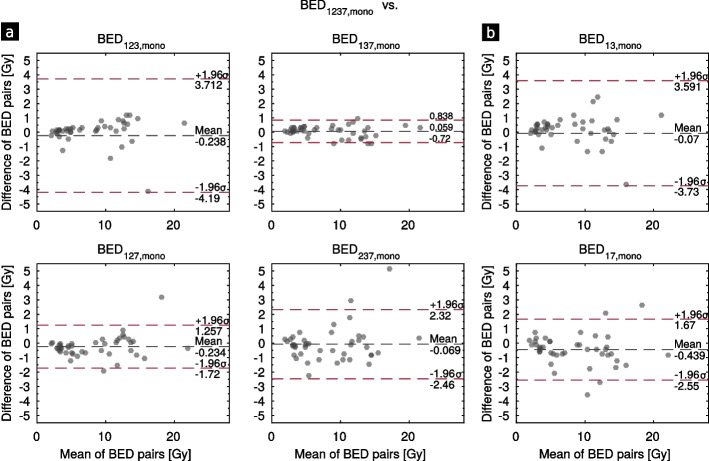
Bland–Altman plots of the different time samplings with 3 TPs (**a**) and 2 TPs (**b**). Please note that for days 1, 2, 3 and 1, 3 there is one outlier lesion with a BED difference of − 11.9 Gy and − 10.4 Gy, respectively. For scaling reasons, they are not shown in these plots but are provided in Additional file 1

**Fig. 4:**
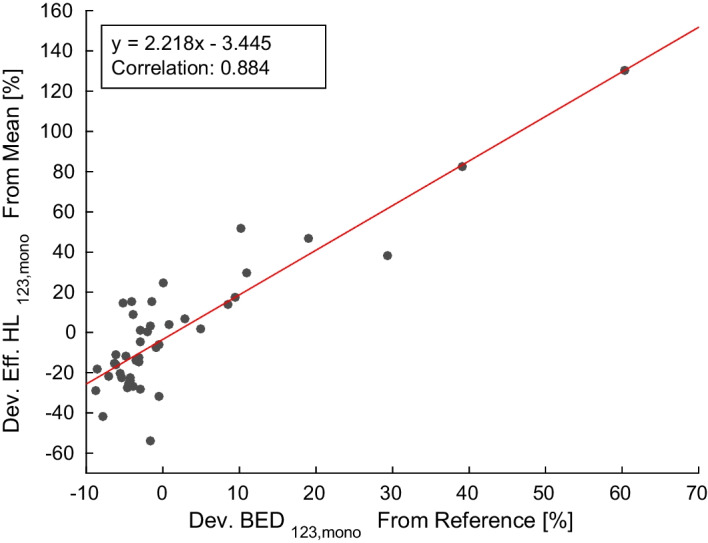
Correlation between the deviation of the BED_123,mono_ from the reference model and the deviation of the effective half-life (HL) from the mean of each patient for all lesions

The deviations of the lesion BEDs of all investigated time samplings comprising 3 and 2 TPs from the reference model BED_1237,mono_ except days 1, 2 (cf. Additional file 1) are visualized by the Bland–Altman plots in Fig. 3. The spread in the differences between the BED value pairs for every lesion is smallest using days 1, 3 and 7. This is also validated by the lowest MD (TPs 1, 3, 7) of 4 ± 5% in Table 3. Further, 95% of all lesion BEDs show a deviation of less than 10% from the reference model. The BED is underestimated in case of approximately 70% of the lesions, when comparing the time samplings for days 1, 2, 3 and 1, 3, 7 and 1, 3 to the reference model. For all other model comparisons, the lesion BED is overestimated. Using only days 1 and 2 for the BED calculation results in large deviations of 49 ± 96% with 37% of the tumors showing deviations smaller than 10%. All deviations for the individual lesions and time samplings are shown in Additional file 1. Considering the BED_123,mono_ calculation, 86% of the lesions deviate less than 10% from the reference model, but there are a few outliers with up to − 60% deviation. For these outliers, the fitted effective half-life is high compared to the effective half-life averaged over all lesions of the respective patient. This correlation is visualized by the plot in Fig. 4, which was fitted by linear regression with the function *y* = 2.218 ± 0.183 *x* − 3.445 ± 2.420. The Pearson correlation coefficient indicates a strong positive correlation of 0.884. The same applies to the time sampling comprising days 1 and 3 with a correlation of 0.783. The TACs of the two outliers of BED_123,mono_ versus reference are provided in Additional file 1.

**Table 3:** Mean lesion BEDs and deviations from the reference model *f*_mono_. Additionally, the percentage of lesions with less than |10| % deviation and only positive deviation from the reference are reported. The latter evaluates how many lesions are overestimated. The mean BED_1237,mono_ (min, max) of the reference model is 8.2 ± 5.0 Gy (2.0 Gy, 21.7 Gy)

Reference versus days	1, 2, 3	1, 3, 7	1, 2, 7	2, 3, 7	1, 2	1, 3	1, 7
Mean BED ± SD (min,max) [Gy]	8.5 ± 5.9 (2.2, 31.6)	8.2 ± 5.1 (1.9, 21.4)	8.5 ± 4.8 (2.1, 22.1)	8.3 ± 4.8 (1.8, 21.4)	12.3 ± 16.1 (2.3, 97)	8.3 ± 5.9 (2.1, 30.1)	8.7 ± 5.1 (1.8, 22.5)
MD ± SD (max) [%]	8 ± 13 (− 60)	4 ± 5 (− 11)	8 ± 9 (− 27)	11 ± 15 (− 52)	49 ± 96 (− 392)	10 ± 14 (− 53)	12 ± 14 (− 47.7)
% lesions w/dev. <|10| %	86	95	67	61	37	65	54
% lesions w/dev. > 0%	30	30	70	62	70	33	72

#### Kidneys

The BED deviations for the kidneys compared to the reference BED_1237,bi,sγ_ are very small, regardless of the time sampling (cf. Table 4). Using TPs 1, 2, 3 days p.i. results in the smallest MD (0 ± 1%) from the reference model, whereas the largest MD with 5 ± 6% was obtained by using 2 TPs with days 1 and 7. The deviations from the reference model of all kidneys and sampling schedules are provided in Additional file 1.

**Table 4:** Resulting median kidney BEDs and mean deviations from the reference model for the different time samplings. The median BED_1237,bi,sγ_ (min,max) of the reference model is 2.0 Gy (1.2 Gy, 2.4 Gy)

Reference versus days	1, 2, 3	1, 3, 7	1, 2, 7	2, 3, 7	1, 2	1, 3	1, 7
Median BED (min,max) [Gy]	2.0 (1.1, 2.4)	2.0 (1.3, 2.4)	2.0 (1.1, 2.4)	2.0 (1.2, 2.4)	2.0 (1.0, 2.4)	2.0 (1.2, 2.4)	2.0 (1.3, 2.5)
MD ± SD (max) [%]	0 ± 1 (− 2)	2 ± 3 (8)	2 ± 3 (− 9)	1 ± 2 (− 5)	3 ± 5 (− 13)	2 ± 2 (6)	5 ± 6 (13)

## Discussion

At the beginning of this study, an evaluation of the NEMA phantom was included. The results confirmed the general feasibility of a short SPECT/CT acquisition time of 5 s per projection over an extended field-of-view with regard to lesion detection and quantification for ^177^Lu-PSMA therapy. Thus, quantitative SPECT/CTs can be acquired over a similar field-of-view as planar whole-body images, but allow for higher quantification accuracy. A volume threshold of 5 ml was defined for a lesion segmentation that ensures robust volume and mass determination. Although the phantom study would allow for a phantom-based recovery correction, the latter was omitted as the true object recovery is highly variable and depends on multiple factors. For example, Tran-Gia et al. showed in their study of ^177^Lu-SPECT/CT-based quantification of a kidney phantom that RCs can strongly depend on the shape of the volume. They found an average difference of 32% between renal and sphere recoveries [35]. Since some of the segmented lesions (e. g. in the pelvis) differ a lot from spheres, recovery correction could introduce a source of uncertainty although it would probably lead to a higher lesion BED.

This study showed that for the investigated cohort of patients, the best-fit function for lesion dosimetry with TPs 1, 2, 3 and 7 days p.i. was found to be a mono-exponential fit. The bi-exponential fit with a shared *γ* parameter visually also worked well but leads to BED results with a MD of 23% from the mono-exponential fit. This stresses the importance of standardized and robust procedures regarding the definition of the best-matching fit function. In this study, the recommendations by Kletting et al. were employed [20]. For the kidneys, the bi-exponential model with a population-based *γ* parameter showed the lowest CVs and SSEs. Our results on the best-matching fit functions for kidney dosimetry agree with the findings of Hardiansyah et al. using their population-based *γ*-parameter [19].

The best time sampling for lesions was the one including days 1, 3, 7 with a MD of only 4%. The standard deviation of 5% was small and the maximum deviation was − 11%. Most lesion BEDs (70%) were underestimated with that time sampling. If no late imaging TP was included, i.e., for the sampling with days 1, 2, 3, the MD and the standard deviation increased up to 8 ± 13% and a maximum deviation of − 60%. However, 86% of the lesions deviated less than ± 10% from the reference. A deeper analysis of the outliers revealed a highly positive correlation (0.884) between the deviation from the reference BED and an untypically high lesion effective half-life compared to the average lesion effective half-life. More precisely, the occurrence of an untypically high lesion effective half-life during the analysis of a patient’s metastases could be associated with a missing late SPECT/CT measurement. Vice versa, an untypically high effective half-life might indicate that the respective dosimetry results without a late SPECT/CT measurement should be handled with care. More statistics would be needed to further investigate this correlation.

For the kidneys, all different investigated time samplings resulted in small deviations (maximum ± 13%) from the bi-exponential reference model with a shared *γ* parameter. The smallest MD was achieved by using TPs 1, 2, 3 days. However, current guidelines recommend three SPECT/CT acquisitions between 1 and 7 days after therapy [8]. The shared parameter regulating the slow pharmacokinetic phase at late TPs after therapy may ensure that the BED is not underestimated if no late TPs are included. Rinscheid et al. determined the best TPs to be 3–4 h, 3 days and 5–6 days p.i. for kidneys alone applying a mono-exponential fit model [36]. The different fit function used in this study may be the reason why a TP later than 3 days p.i. is not important for the cohort in this study. For lesions, this study also showed that a late TP is more important for dosimetry, since the schedule with days 1, 3, 7 leads to a lower standard deviation than the one with days 1, 2, 3. Accordingly, Rinscheid et al. found out that in case of joint optimization for kidney and lesion dosimetry, a later TP becomes important and the optimal sampling changes to 3–4 h, 4 days and 8 days p.i. for three TPs and 1 and 8 days for two TPs [18].

Generally, sampling schedules comprising three and two TPs were chosen in this study in combination with fit functions including two free parameters which is not optimal from a methodological perspective. More data points would be beneficial since fitting a model with two free parameters to two TPs contradicts the general rule of *N* − *K* > 0 (*N*: number of data points; *K*: number of free parameters) [8]. However, due to varying clinical capability amongst hospitals, a varying patient catchment area or different radiation protection regulations different sampling schedules may be applicable, especially regarding late acquisition time points. This limits the choice of possible suitable fit models. For lesions, this would motivate to include a population-based effective half-life or further approximations into the mono-exponential model as it has already been done in single TP dosimetry [37, 38].

The found deviations of all investigated time samplings can be considered to be of minor impact compared to an error of 30 to 100%, that has to be expected for internal dosimetry in general [39]. Gustafsson et al. specifically investigated the impact of different sources of uncertainties in the dosimetric process on renal BED after ^177^Lu-DOTATATE therapy with a Monte Carlo approach. Variabilities were added to the different steps of the process like the gamma-camera calibration, the CT density, SPECT reconstruction, VOI segmentation, recovery correction and the calculation of the BED. Their impact on the total uncertainty in BED was evaluated by removing different sources of uncertainties. One of the sources with the highest propagated uncertainty in BED was found to be the recovery correction with a systematic uncertainty of 15% and a doubled standard deviation compared to the reference. The standard deviation of the BED using the total model was reported to be approximately 6% [40]. Systematic analysis of all sources of errors for studies on ^177^Lu-PSMA dosimetry would be of interest as suggested by Gear et al. [41]. Due to the high number of sources of uncertainties, every step of the dosimetry process should be performed as accurate as possible. However, patient compliance and well-being are important factors within the therapeutic procedure and access to dosimetry shall be available for all patients. Thus, a reasonable number of imaging TPs along with the uncertainty that has to be expected for the employed sampling schedule is highly desirable.

In this study, only five patients were included which generally can be considered as a low sample size for statistical purposes. The availability of four scan TPS including a late measurement at day 7 p.i. limits the size of the patient cohort. However, since all acquisitions were SPECT/CT measurements, at least 43 lesions could be segmented and evaluated.

## Conclusion

For lesions, the time sampling including 1, 3, 7 days p.i. with three TPs showed the lowest deviations from the reference (4 ± 5%), i.e., a mono-exponential fit to days 1, 2, 3 and 7. If the late measurement TP at day 7 shall be avoided with the sampling schedule 1, 2, 3 days, still 86% of the lesions showed a deviation less than 10% from the reference. The observed deviations for that sampling schedule show a strong correlation with the deviation of the effective lesion half-life from the mean patient-specific average effective lesion half-life. The deviations for kidneys were lower in general, with the best time sampling at days 1, 2, 3 for three TPs and 1, 3 for two TPs (0 ± 1% and 2 ± 2%).

## Supplementary Information

**Additional file 1.** Supplementary data plots as referenced in the main text.

## Data Availability

Please contact the corresponding author.
