# Supplementary material for: Investigation of image-based lesion and kidney dosimetry protocols for 177Lu-PSMA-I&T therapy with and without a late SPECT/CT acquisition
Source: EJNMMI Phys. 2023 Feb 9;10:11. doi: 10.1186/s40658-023-00529-8 (PMC9911578; doi:10.1186/s40658-023-00529-8)
Supplement: Supplementary file 1 — Additional file 1. Supplementary data plots as referenced in the main text. [file 40658_2023_529_MOESM1_ESM.docx]

*Supplemental* *File*

*
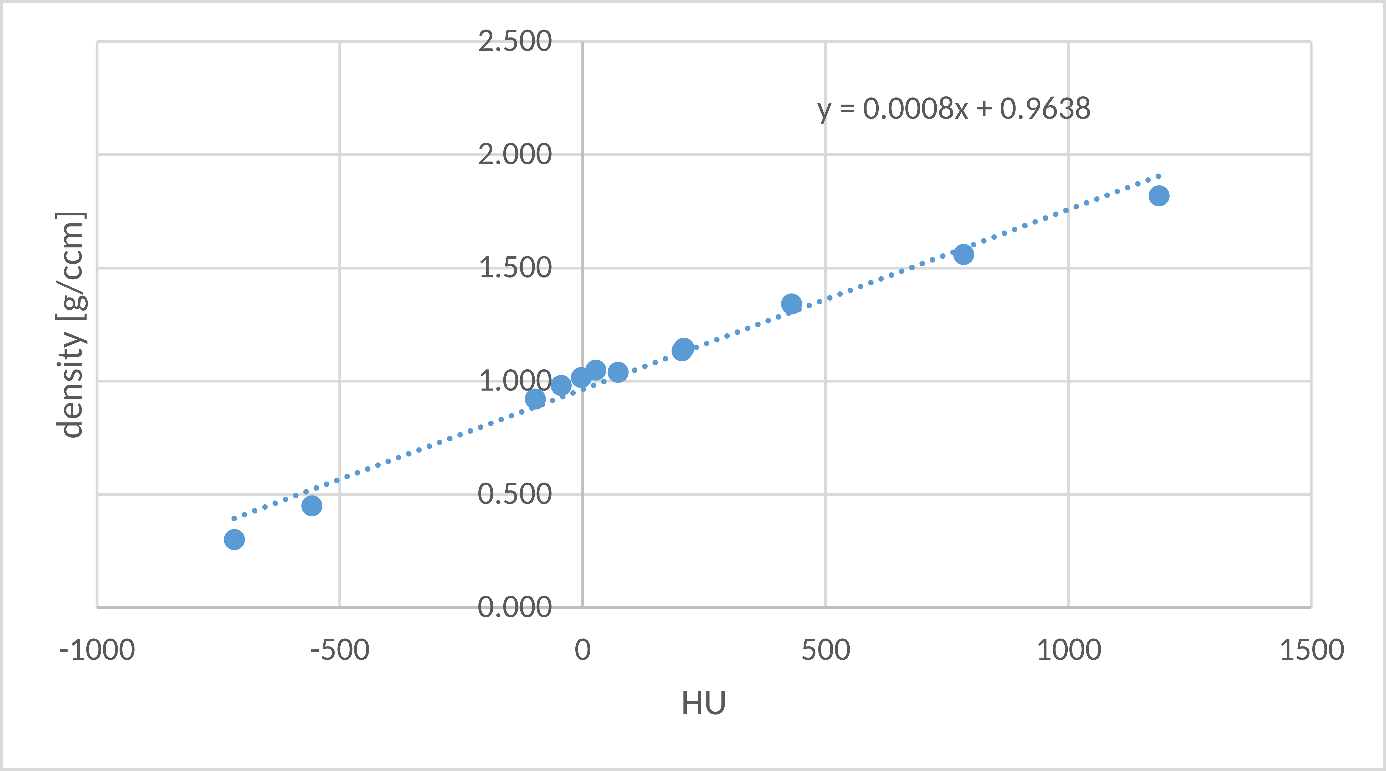
*

**Figure A 1:** HU-density plot with linear fit.


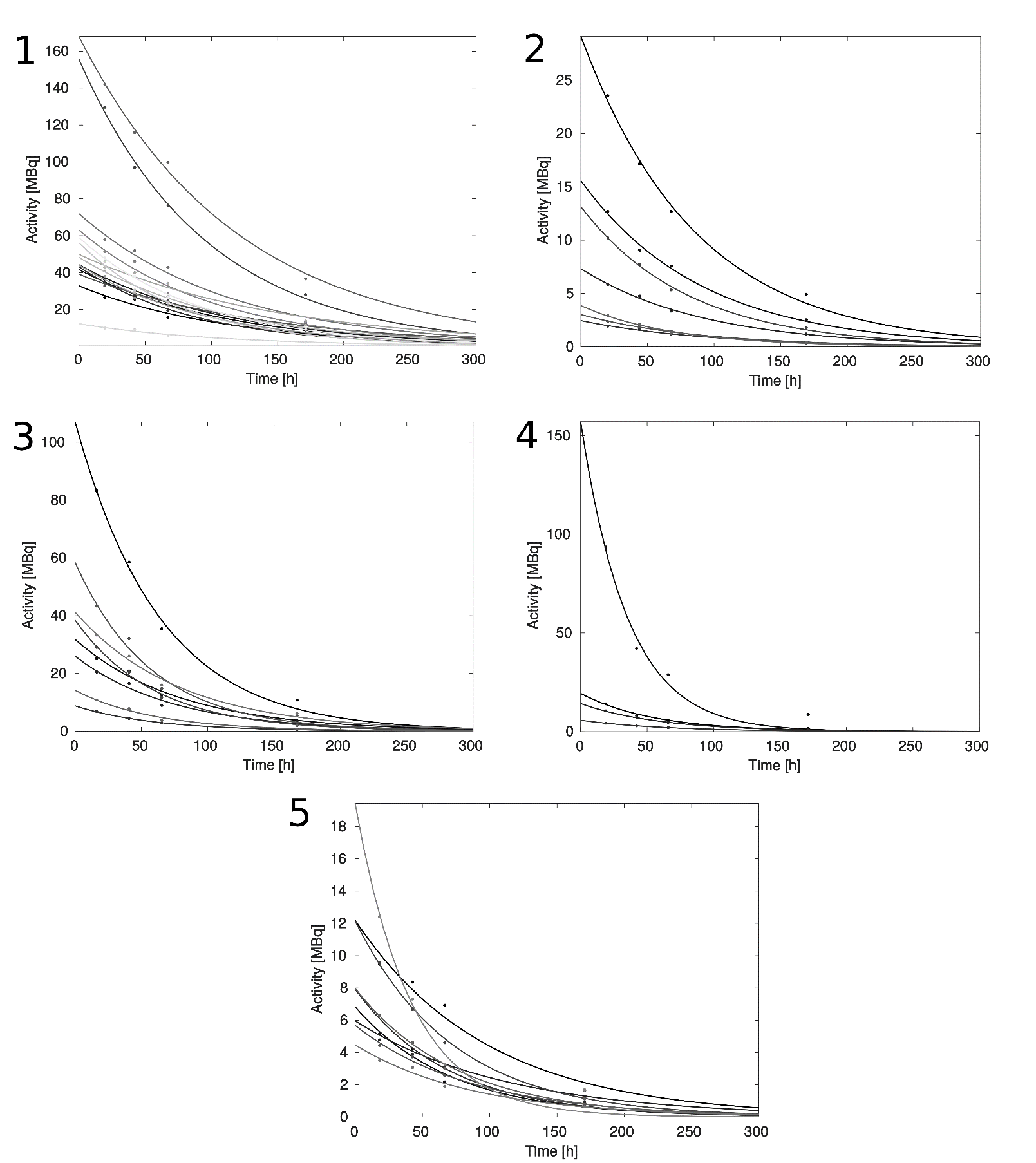


**Figure A 2:** TACs of all lesions for patients 1 to 5. The curves were fitted including all four data points at days 1, 2, 3 and 7 and using a mono-exponential model representing the reference model.


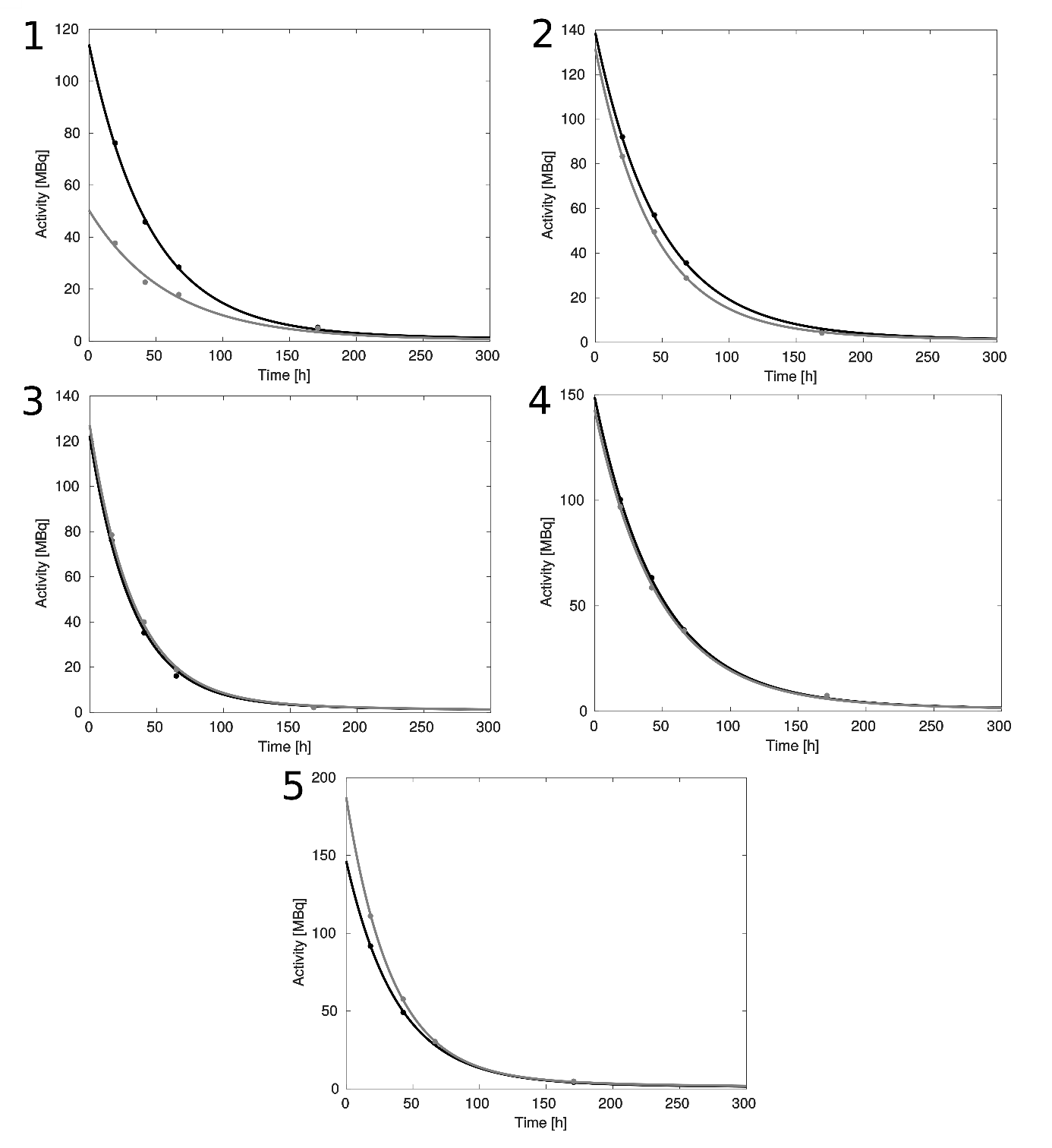


**Figure A 3:** TACs of all kidneys for patients 1 to 5. The curves were fitted including all four data points at days 1, 2, 3 and 7 and using a bi-exponential model with a population-based parameter representing the reference model.


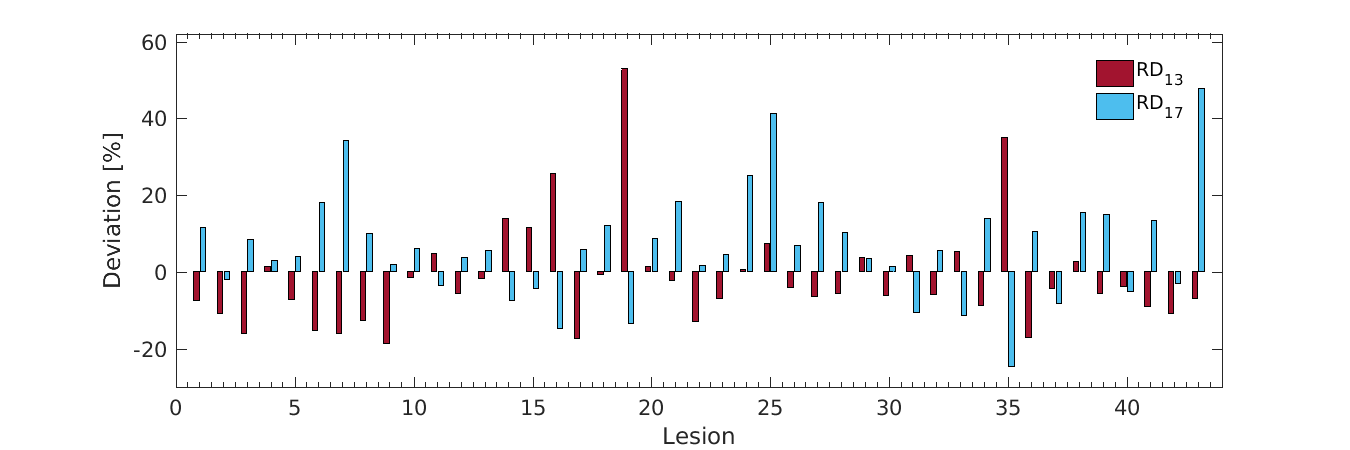

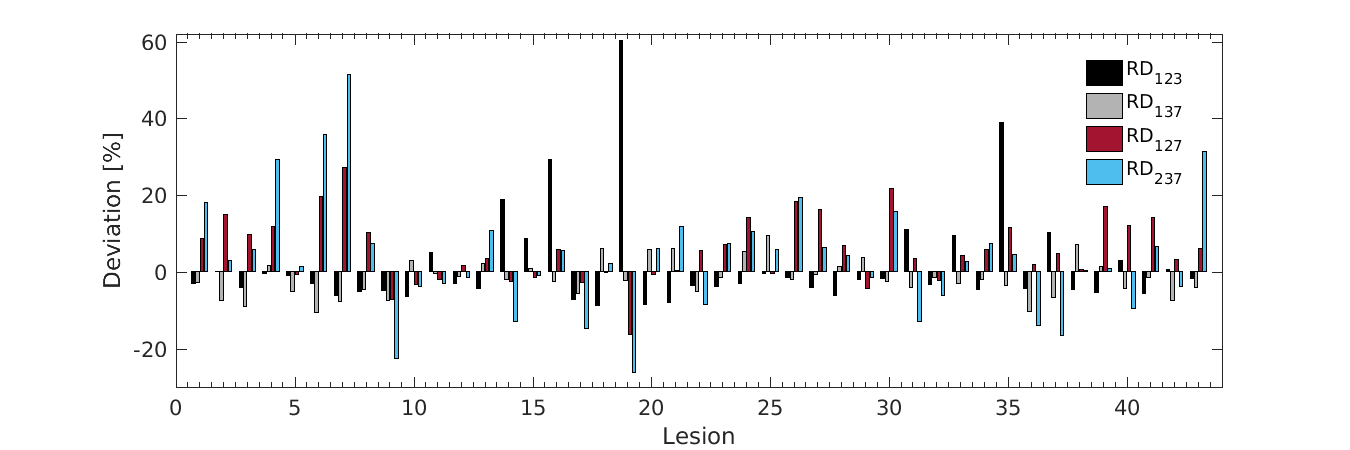


**Figure A 4:** Deviations of the BEDs from the reference function for every lesion. The upper plot shows the deviations for three measurement time points, the lower plot for two time points. The deviations with the sampling schedule 1, 2 days are not shown here since the deviations are out the presented range (max. -392 %).


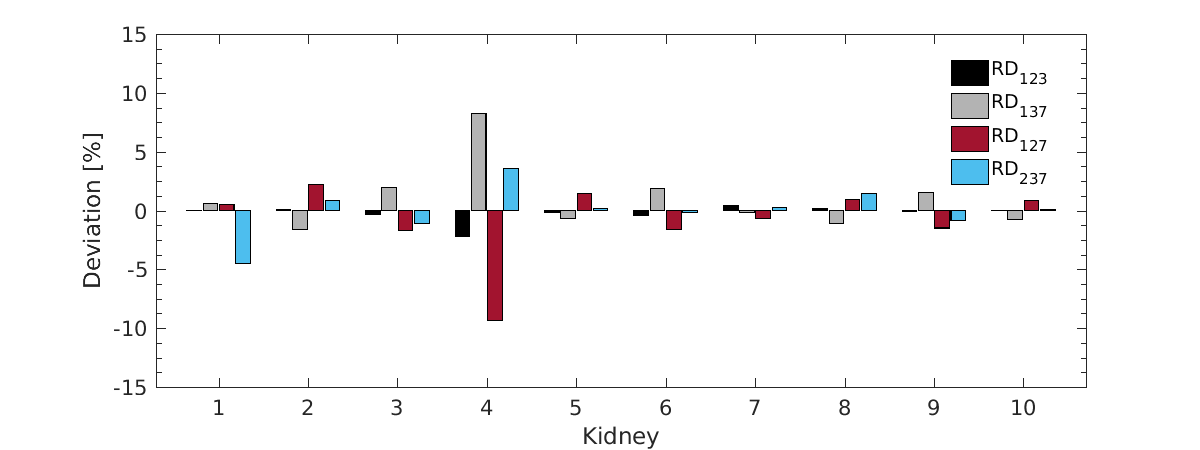

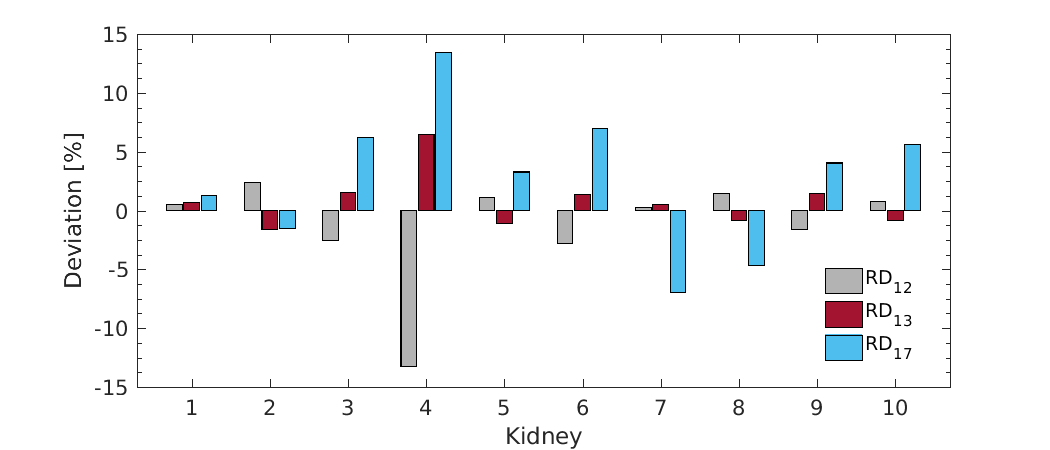


**Figure A 5:** BED deviations from the reference function for all kidneys for three time points (upper plot) and two time points (lower plot).


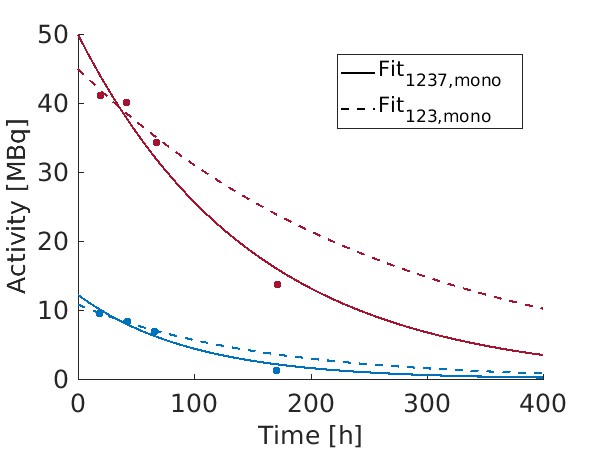


**Figure A 6:** TACs of the outlier deviations BED_123,mono_ from the reference.


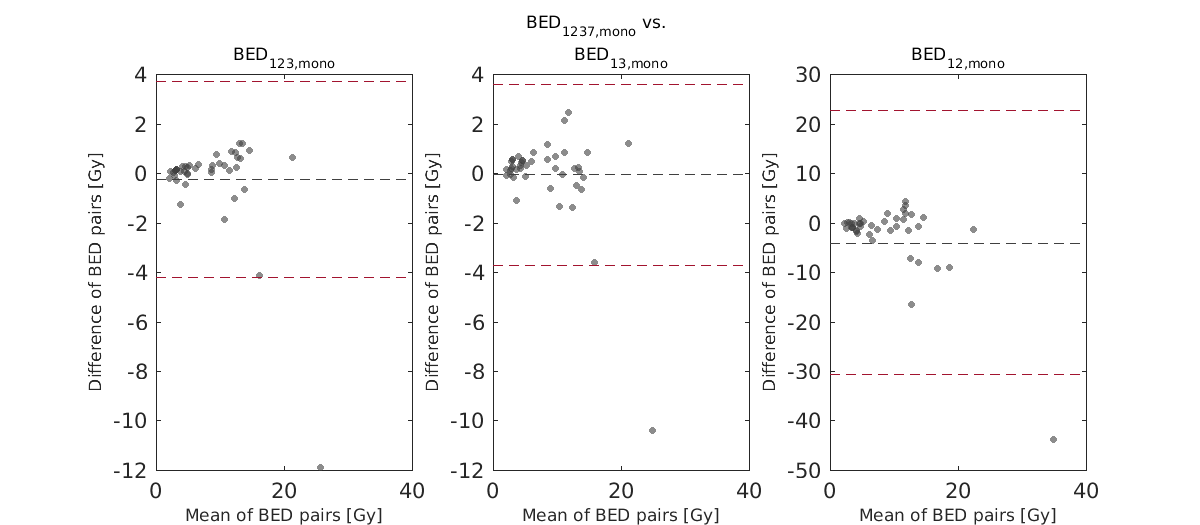


**Figure A 7:** Complete and missing Bland-Altman plots for lesions.
